# Effects of anterior cingulate cortex lesions on a continuous performance task for mice

**DOI:** 10.1177/2398212818772962

**Published:** 2018-05-29

**Authors:** Martha Hvoslef-Eide, Simon R. O. Nilsson, Jonathan M. Hailwood, Trevor W. Robbins, Lisa M. Saksida, Adam C. Mar, Timothy J. Bussey

**Affiliations:** 1 Department of Psychology, University of Cambridge, Cambridge, UK; 2 MRC and Wellcome Trust Behavioural and Clinical Neuroscience Institute, University of Cambridge, Cambridge, UK; 3 Department of Biosciences, University of Oslo, Oslo, Norway; 4 Neuroscience Institute, New York University Medical Center, New York, NY, USA; 5 Department of Neuroscience and Physiology, New York University Medical Center, New York, NY, USA; 6 Molecular Medicine Research Group, Robarts Research Institute, Western University, London, ON, Canada; 7 Department of Physiology and Pharmacology, Schulich School of Medicine & Dentistry, Western University, London, ON, Canada; 8 The Brain and Mind Institute, Western University, London, ON, Canada

**Keywords:** Executive function, touchscreen, animal model, mouse, prefrontal cortex, anterior cingulate cortex, continuous performance task

## Abstract

**Background:**

Important tools in the study of prefrontal cortical -dependent executive functions are cross-species behavioural tasks with translational validity. A widely used test of executive function and attention in humans is the continuous performance task. Optimal performance in variations of this task is associated with activity along the medial wall of the prefrontal cortex, including the anterior cingulate cortex, for its essential components such as response control, target detection and processing of false alarm errors.

**Methods:**

We assess the validity of a recently developed rodent touchscreen continuous performance task that is analogous to typical human continuous performance task procedures. Here, we evaluate the performance of mice with quinolinic acid -induced lesions centred on the anterior cingulate cortex in the rodent touchscreen continuous performance task following a range of task parameter manipulations designed to challenge attention and impulse control.

**Results:**

Lesioned mice showed a disinhibited response profile expressed as a decreased response criterion and increased false alarm rates. Anterior cingulate cortex lesions also resulted in a milder increase in inter-trial interval responses and hit rate. Lesions did not affect discriminative sensitivity d′. The disinhibited behaviour of anterior cingulate cortex -lesioned animals was stable and not affected by the manipulation of variable task parameter manipulations designed to increase task difficulty. The results are in general agreement with human studies implicating the anterior cingulate cortex in the processing of inappropriate responses.

**Conclusion:**

We conclude that the rodent touchscreen continuous performance task may be useful for studying prefrontal cortex function in mice and has the capability of providing meaningful links between animal and human cognitive tasks.

## Introduction

The prefrontal cortex is a functionally heterogeneous region supporting several interconnected ‘executive’ cognitive processes that serve to monitor action-outcome associations and optimise goal-directed action (Dalley et al., 2004). It is widely acknowledged that such prefrontal cortical–dependent functions comprise response control and attentional processes (Robbins et al., 1996; Sarter et al., 2001) that support performance in challenging situations. Deficits in these functions are detectable in individuals with neuropsychiatric disorders through highly standardised and automated tests of cognition (Barch et al., 2009), but the aetiology of these disorders remain incompletely understood and the deficits are poorly treated (Insel et al., 2013; Millan et al., 2012). A standard assessment paradigm of attentional and response control in clinical and human experimental studies has been the continuous performance task (CPT; Rosvold et al., 1956) combined with signal detection analysis (Green and Swets, 1966). In such tests, subjects are exposed to a stream of continuously presented complex non-spatial stimuli. Rapid stimulus processing and response control are required to detect target and non-target stimuli, and to initiate and inhibit inappropriate responding accordingly. These tasks have been used successfully to identify genetic and neural mechanisms of relevance for cognitive function and approaches to cognitive enhancement in humans (Carter et al., 1998; Cornblatt et al., 2003; Rubia et al., 2001; Seidman et al., 2015).

Theoretical accounts postulate critical roles of the anterior cingulate in inhibitory and attentional control (Corbetta and Shulman, 2002; Posner and Petersen, 1990; Stuss et al., 1995). Human imaging and electrophysiological studies identify roles for the anterior cingulate in diverse processes, including response inhibition and the monitoring of conflict and response errors, in order to support behavioural adaptation and sustaining performance under demanding conditions (Botvinick et al., 2004). As assessed in CPTs and Go/No-Go tasks, the anterior cingulate supports the processing of false alarm errors and response inhibition (Botvinick et al., 2004; Casey et al., 2008; Fallgatter et al., 2001). Disrupted anterior cingulate activity is also associated with disinhibited responding, increased false alarm error and impaired discrimination in individuals with prefrontal cortical lesions (Glosser and Goodglass, 1990; Salmaso and Denes, 1982) or diagnosed with psychiatric disorders (Fallgatter et al., 2003; Hester and Garavan, 2004; Leland et al., 2008).

Several rodent analogues of the human CPT, some amenable to signal detection analysis, have successfully been developed with the aim of identifying loci of executive functioning and targets with translational value (Carli et al., 1983; McGaughy and Sarter, 1995; Young et al., 2009). In translational agreement with human studies, this work demonstrates that performances are related to activity along the medial wall of the prefrontal cortex in the rodent using localised lesions (Muir et al., 1996), site-specific pharmacological injections (Murphy et al., 2011; Paine et al., 2011; Pehrson et al., 2013; Pezze et al., 2014), electrophysiological measures (Totah et al., 2009, 2013), optogenetics (Kim et al., 2016), chemogenetics (Koike et al., 2016) and neurochemical correlates (Barbelivien et al., 2001; Dalley et al., 2002; Jupp et al., 2013). In rodent operant assays, anterior cingulate cortex (ACC) activity appears particularly linked to motor impulsivity with manipulations affecting measures such as premature responses and/or response inhibition or approaches to non-target stimuli in detection and discrimination tasks (Bussey et al., 1997; Jupp et al., 2013; Muir et al., 1996; Totah et al., 2009). Others have also found that ACC lesions in the rat can disrupt attention as measured by discriminative sensitivity (Passetti et al., 2002) and impair set-shifting as well as the processing of irrelevant stimuli (Ng et al., 2007).

Yet while rodent behavioural analogues of human CPTs often employ detection of auditory or visual stimuli, human CPT paradigms generally employ visual discrimination tasks that include identification of (a) multiple complex luminance-matched visual stimuli and (b) multiple non-target stimuli, occurring at a single response location. Extant spatial, auditory or visuospatial rodent paradigms employ some, but not all, of these features. There is good evidence that different neural and perceptual/cognitive processes may be recruited because of such cross-species task differences (Lashley, 1931; Petruno et al., 2013; Pöppel et al., 1973; Stoerig et al., 1985) that may contribute to decreased validity, translational difficulties and ultimately attrition of therapeutic candidates (Tricklebank and Garner, 2012).

The rodent touchscreen operant chamber provides an opportunity for the back-translation of standard human CPT procedures into highly analogous rodent testing protocol. In recent reports, we developed a novel rodent touchscreen version of the CPT (rodent CPT or rCPT – Kim et al., 2015; Mar et al., 2017). C57BL/6J and DBA/2J mice were demonstrated to readily acquire the rCPT, with strain differences in task performance observed following manipulations of key task parameters and following donepezil administration (Kim et al., 2015). The rat mitotic neurotoxin methylazoxymethanol acetate model (MAM-E17) of schizophrenia has also been demonstrated to have robust and persistent impairments on measures of attentional control and executive function in the rCPT (Mar et al., 2017). This study, in parallel with ongoing studies assessing the functional heterogeneity of the rat prefrontal cortex in the rCPT (Mar et al., unpublished; Fisher et al., unpublished), aims to further validate the rCPT by establishing the degree to which task performance in the mouse depends on activity in the prefrontal cortex. As part of this work, the current study tested the hypothesis that the mouse anterior cingulate is important for rCPT performance. Here, we evaluate the performance of mice with excitotoxic lesions centred on the anterior cingulate and sham-lesioned controls in the rCPT. Animals were tested following several task parameter manipulations designed to challenge performance further (Kim et al., 2015).

## Methods

### Animals

In total, 32 male C57BL/6J mice (Charles River, UK) started behavioural testing at 7–9 weeks of age. Animals were group-housed under a 12-h light/dark cycle (lights on at 7:00 a.m.) with stable temperature and humidity conditions with ad libitum access to food and water. Experiments were carried out during the dark phase of the light cycle. Prior to the start of testing, animals were food restricted and maintained at 85%–90% of their free-feeding body weights. Neophobia to the test diet (14 mg Bio-Serv purified rodent dustless precision pellets; Sandown Scientific, Middlesex, UK) was reduced by exposure in the home cage prior to operant training. This research has been regulated under the Animals (Scientific Procedures) Act 1986 Amendment Regulations 2012 following ethical review by the University of Cambridge Animal Welfare and Ethical Review Body (AWERB). Two animals unexpectedly died towards the end of the study but were included in the analysis where their data were complete. In all, 10 animals were omitted from the analyses. This was due to failure to reach the performance criterion pre-surgery (n = 2), complications following surgery (n = 2), injury from post-surgery fighting (n = 2) and unexpected death early in the study (n = 4). The exact n numbers for each group are in Table 1.

**Table 1.** Mean values ± SEM for sham and ACC-lesioned mice in each probe and on two averaged baseline sessions immediately prior to the start of post-surgery probes.

	c		FAR		HR		d′	
	Sham	Lesion	Sham	Lesion	Sham	Lesion	Sham	Lesion
Baseline (4 s SD; n = 10; 14)	0.46 ± 0.05	0.35 ± 0.10	0.19 ± 0.02	0.23 ± 0.03	0.50 ± 0.02	0.53 ± 0.04	0.91 ± 0.05	0.84 ± 0.08
vSD#1 (s) (n = 10; 14)								
4	**0.56 ± 0.07**	**0.25 ± 0.11**	**0.17 ± 0.02**	**0.27 ± 0.04**	0.45 ± 0.03	0.56 ± 0.04	0.88 ± 0.08	0.82 ± 0.11
3	**0.60 ± 0.05**	**0.36 ± 0.10**	**0.19 ± 0.02**	**0.27 ± 0.04**	0.40 ± 0.02	0.48 ± 0.04	0.64 ± 0.07	0.59 ± 0.09
2	**0.55 ± 0.05**	**0.41 ± 0.12**	**0.24 ± 0.02**	**0.31 ± 0.04**	0.37 ± 0.02	0.40 ± 0.04	0.41 ± 0.06	0.25 ± 0.09
1	**0.64 ± 0.04**	**0.34 ± 0.12**	**0.24 ± 0.02**	**0.35 ± 0.04**	0.31 ± 0.02	0.41 ± 0.04	0.20 ± 0.07	0.17 ± 0.05
vSD#2 (s) (n = 10; 14)								
1	0.63 ± 0.05	0.57 ± 0.18	0.26 ± 0.02	0.30 ± 0.05	0.29 ± 0.02	0.33 ± 0.06	0.11 ± 0.05	0.08 ± 0.09
0.75	0.64 ± 0.04	0.53 ± 0.17	0.26 ± 0.01	0.32 ± 0.05	0.28 ± 0.02	0.34 ± 0.05	0.05 ± 0.07	0.07 ± 0.06
0.5	0.62 ± 0.05	0.64 ± 0.19	0.27 ± 0.02	0.29 ± 0.05	0.28 ± 0.02	0.30 ± 0.05	−0.01 ± 0.07	0.06 ± 0.06
0.25	0.61 ± 0.04	0.60 ± 0.20	0.28 ± 0.01	0.34 ± 0.05	0.27 ± 0.02	0.28 ± 0.05	−0.01 ± 0.05	−0.16 ± 0.10
vSD#3 (s)								
(n = 7; 14) 3	**0.66 ± 0.05**	**0.49 ± 0.06**	**0.17 ± 0.03**	**0.23 ± 0.03**	0.39 ± 0.03	0.42 ± 0.04	0.74 ± 0.15	0.55 ± 0.17
2	**0.71 ± 0.05**	**0.54 ± 0.05**	**0.18 ± 0.02**	**0.22 ± 0.02**	0.33 ± 0.02	0.41 ± 0.03	0.54 ± 0.13	0.60 ± 0.10
1	**0.74 ± 0.05**	**0.55 ± 0.09**	**0.20 ± 0.02**	**0.26 ± 0.03**	0.28 ± 0.02	0.34 ± 0.03	0.29 ± 0.11	0.24 ± 0.11
vSD#4 (s)								
(n = 6; 14) 5	**0.45 ± 0.04**	**0.27 ± 0.09**	**0.23 ± 0.02**	**0.30 ± 0.04**	0.50 ± 0.06	0.47 ± 0.03	0.71 ± 0.13	0.59 ± 0.23
3	**0.50 ± 0.05**	**0.28 ± 0.08**	**0.23 ± 0.02**	**0.34 ± 0.05**	0.45 ± 0.04	0.42 ± 0.03	0.56 ± 0.11	0.31 ± 0.17
1	**0.53 ± 0.06**	**0.35 ± 0.08**	**0.29 ± 0.02**	**0.39 ± 0.05**	0.35 ± 0.03	0.32 ± 0.02	0.11 ± 0.07	−0.12 ± 0.13
Fixed SD (s) (n = 6; 14)								
5	0.45 ± 0.06	0.31 ± 0.10	0.23 ± 0.02	0.27 ± 0.03	0.46 ± 0.03	0.51 ± 0.07	0.70 ± 0.12	0.70 ± 0.22
1	1.11 ± 0.04	1.02 ± 0.07	0.08 ± 0.01	0.11 ± 0.01	0.22 ± 0.01	0.23 ± 0.04	0.68 ± 0.07	0.54 ± 0.11
S+ probability (%)(n = 8; 14)								
50	**0.70 ± 0.05**	**0.45 ± 0.11**	**0.16 ± 0.01**	**0.24 ± 0.04**	0.36 ± 0.02	0.41 ± 0.05	0.66 ± 0.08	0.60 ± 0.12
30	**0.73 ± 0.04**	**0.54 ± 0.07**	**0.15 ± 0.01**	**0.21 ± 0.03**	0.36 ± 0.03	0.41 ± 0.04	0.71 ± 0.12	0.60 ± 0.12
ITI (s) (n = 8; 14)								
2	**0.70 ± 0.05**	**0.45 ± 0.11**	**0.16 ± 0.01**	**0.24 ± 0.04**	0.36 ± 0.02	0.44 ± 0.05	0.66 ± 0.08	0.60 ± 0.12
4	**0.78 ± 0.03**	**0.61 ± 0.10**	**0.13 ± 0.02**	**0.18 ± 0.03**	0.36 ± 0.03	0.41 ± 0.05	0.82 ± 0.17	0.76 ± 0.16
Length (min) (n = 6; 14)								
45	1.11 ± 0.04	1.02 ± 0.07	0.08 ± 0.01	0.11 ± 0.01	0.22 ± 0.01	0.23 ± 0.04	0.68 ± 0.07	0.54 ± 0.11
90	1.06 ± 0.03	0.89 ± 0.07	0.09 ± 0.01	0.13 ± 0.02	0.24 ± 0.01	0.27 ± 0.03	0.65 ± 0.08	0.52 ± 0.09
Distractors#1 (4 s) (n = 10; 14)								
None	0.72 ± 0.07	0.49 ± 0.10	0.16 ± 0.02	0.23 ± 0.05	**0.39 ± 0.02**	**0.46 ± 0.02**	0.83 ± 0.10	0.75 ± 0.17
Congruent	1.00 ± 0.07	0.72 ± 0.16	0.09 ± 0.01	0.16 ± 0.04	**0.30 ± 0.02**	**0.39 ± 0.05**	0.89 ± 0.09	0.86 ± 0.12
Incongruent	0.98 ± 0.07	0.69 ± 0.13	0.09 ± 0.01	0.16 ± 0.04	**0.32 ± 0.02**	**0.40 ± 0.05**	1.00 ± 0.07	0.82 ± 0.11
Distractors#2 (2.5 s) (n = 9; 14)								
None	0.56 ± 0.05	0.42 ± 0.09	0.20 ± 0.02	0.27 ± 0.03	0.41 ± 0.02	0.43 ± 0.04	0.64 ± 0.09	0.46 ± 0.06
Congruent	0.80 ± 0.06	0.67 ± 0.07	0.14 ± 0.01	0.17 ± 0.01	0.33 ± 0.02	0.37 ± 0.04	0.73 ± 0.07	0.64 ± 0.11
Incongruent	0.66 ± 0.09	0.53 ± 0.10	0.19 ± 0.02	0.21 ± 0.03	0.36 ± 0.03	0.42 ± 0.04	0.56 ± 0.05	0.63 ± 0.07
Distractors#3 (1 s) (n = 10; 13)								
None	0.36 ± 0.08	0.25 ± 0.08	0.37 ± 0.09	0.32 ± 0.09	0.44 ± 0.09	0.44 ± 0.14	0.22 ± 0.09	0.40 ± 0.11
Congruent	0.22 ± 0.05	0.27 ± 0.06	0.24 ± 0.04	0.28 ± 0.06	0.26 ± 0.03	0.35 ± 0.04	0.40 ± 0.09	0.29 ± 0.08
Incongruent	0.56 ± 0.08	0.31 ± 0.11	0.39 ± 0.07	0.30 ± 0.12	0.40 ± 0.07	0.35 ± 0.07	0.35 ± 0.08	0.30 ± 0.09

P

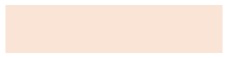
 <0.05 Significant effect of lesion

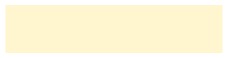
 <0.025

ACC: anterior cingulate cortex; c: response criterion; FAR: false alarm rate; HR: hit rate; d′: discrimination sensitivity; SD: stimulus duration; vSD: variable stimulus durations; ITI: inter-trial interval.

Significant main effects of lesion are in bold and colour (see legend). Interaction effects between group and probe are denoted by colour only. N numbers are listed in the order ‘lesion’, followed by ‘sham’.

### Apparatus

Testing was conducted in modified Med Associates, Inc. (St. Albans, VT, USA) touchscreen operant chambers for mice as described elsewhere (Horner et al., 2013; Mar et al., 2013) controlled by in-house software (Visual Basic 2010 Express .NET, Microsoft 2010; developed by A.C.M.). In brief, the apparatus consisted of a rectangular chamber with an infrared touchscreen at one end and a reward magazine (with a photocell head entry detector) illuminated by a 3 W light bulb at the other end. A three-aperture mask (Kim et al., 2015) covered the touchscreen. The walls were clear Perspex with a metal grid floor. The chamber was housed within a sound attenuating box fitted with a fan for ventilation and masking of external noise, a pellet dispenser delivering reward pellets and a tone generator.

### Procedure

#### Pre-surgery training

The training procedure is described elsewhere (Kim et al., 2015). In brief, animals were trained in four stages. In *Stage 1*, a trial started with the onset of a white square stimulus (3.5 cm × 3.5 cm) within a centrally located white frame on the touch-sensitive screen. The stimulus duration (SD) was 10 s, with a 2-s inter-trial interval (ITI, initiated at reward collection) and a limited hold (LH) of 10.5 s (i.e. responses were recorded at 0.5 s after the removal of the stimulus from the screen to account for responses initiated late during the stimulus presentation). A response to the stimulus within the LH resulted in stimulus removal, a 1-s tone, illumination of the magazine light and reward delivery. A session either terminated after 45 min or after 80 rewards had been collected. Throughout all testing, touches to the empty white frame during the ITI (‘ITI touch’) resulted in re-setting the ITI timer, thereby delaying the presentation of the next stimulus. When reaching the criterion of 60 responses to the stimulus (i.e. 60 rewards) in a 45-min session, Stage 2 was introduced. In *Stage 2*, the target stimulus (S+) was presented (horizontal lines or vertical lines; counterbalanced across animals) and the SD was reduced to 4 s (LH = 4.5 s). After a response to the stimulus, a short extension of the ITI was introduced (‘ingestion delay’; 5 s) to allow the animal to consume the reward. No other parameters were changed from Stage 1. The session lasted for 45 min or 60 rewards, whichever occurred first. The criterion for progressing to Stage 3 was 60 rewards in a single session. In *Stage 3*, animals were presented with the S+ on 50% of the trials and a novel unrewarded stimulus (‘snowflake’, S–; see Kim et al., 2015) on 50% of the trials. If the animal responded to the S–, the stimulus was removed, the ITI was initiated and the next trial was a correction trial (in which the S– was presented repeatedly until the animal withheld a response). Animals were trained for at least eight sessions on Stage 3, and the performance criterion for moving on to the baseline rCPT procedure was a discriminative sensitivity (d′; see ‘Data analyses and statistics’ section) above 0.6. In the *baseline rCPT*, the ‘snowflake’ stimulus was replaced with four novel S– stimuli (see Kim et al., 2015). On a given trial, the probability of the S+ stimulus being presented was 50%, with one of the four S– stimuli being presented on the remaining 50% of trials (in addition to correction trials, which were exclusively S– trials). No other parameters were changed between stage 3 and the baseline rCPT. Animals were trained on the baseline rCPT for a minimum of four sessions and the criterion for progressing was a d′above 0.6. When criterion had been achieved, animals were exposed to rCPT probes both before and after quinolinic acid–induced lesions.

#### Pre-surgery probe testing

Some experiments have emphasised a role for the prefrontal cortex and the ACC in novelty processing (Berns et al., 1997; Weible et al., 2009; Zhu et al., 1995). In order to reduce possible interactions between test novelty and ACC lesioning on measures of attention and inhibitory control, mice had pre-surgery exposure to probe tests, run in a similar manner to the critical post-surgery tests, for one session per probe after reaching criterion on the baseline rCPT. These included reduction in SD, increased ITI, lowered target (S+) probability and the presence of flanking distractors.

#### Surgery

Mice were placed in a stereotaxic frame (Kopf Instruments, Tujunga, CA, USA) under constant isoflurane gas anaesthesia. Following a midline incision of the skin, a flat skull surface was ensured prior to the drilling burr holes above injection sites (anterior-posterior axis (AP) +2.0, medial-lateral axis (ML) ±0.3 and dorsal-ventral axis (DV) −2.5; from Dura). For the lesion group, 0.4 µL of 60 mM quinolinic acid (2,3-Pyridinedicarboxylic acid, P3504-10G; Sigma-Aldrich, Gillingham, UK) in 0.1 M phosphate buffered saline (PBS) was infused at a rate of 0.1 µL/min; 5 min passed prior to raising the needle to ensure dispersion from the infusion site. For the sham surgery control group, the injector was lowered to the same coordinate as the lesion group, but nothing was infused. All animals were treated with a peripheral analgesic post-surgery (0.05 mg meloxicam, i.p.; Boehringer Ingelheim, Bracknell, UK). Animals were returned to food restriction and behavioural testing following full recovery from surgery.

#### Post-surgery probe testing

After surgery recovery, all mice were tested on the baseline rCPT parameters until reaching a d′ of 0.6 for one session. The animals were then tested on a series of probe tasks designed to create challenging task conditions. In these probe tests, we systemically varied single task parameter while other parameters remained constant. These task manipulations have previously been used to gauge attentional functions in human studies (Berwid et al., 2005; Cattapan-Ludewig et al., 2005; Conners et al., 2003; Davies and Parasuraman, 1982; Epstein et al., 2007; Mass et al., 2000; Parasuraman, 1979; Rose et al., 2001; Stroh, 1971). The probe tests were presented in the order they are listed in Table 2.

**Table 2.** ITI touches by lesioned animals and sham controls in rCPT testing.

	Sham	Lesion
rCPT baseline 4 s SD	265 ± 19	393 ± 51*
vSD#1	267 ± 27	389 ± 52*
vSD#2	364 ± 28	512 ± 124
Distractors#1	168 ± 27	290 ± 67
Distractors#2	353 ± 42	427 ± 63
Distractors#3	443 ± 42	447 ± 49
S+ probability	344 ± 30	424 ± 90
ITI	337 ± 37	389 ± 69
vSD#3	272 ± 35	381 ± 54
vSD#4	279 ± 21	396 ± 93
Fixed SD 1 s	187 ± 23	265 ± 27
Fixed SD 5 s	186 ± 18	259 ± 41
Session length (90 min)	615 ± 59	973 ± 123**

ITI: inter-trial interval; rCPT: rodent continuous performance task; SD: stimulus duration; vSD: variable stimulus durations.

* p < 0.05; **p < 0.01.

##### Manipulating SDs

We introduced variable stimulus durations (vSD) based on the prediction that shorter SDs place greater demand on attentional processes through limited detection times (Mass et al., 2000; Parasuraman and Davies, 1984). We tested animals on four tests where vSD spanned different ranges. The different SDs were presented with an equal and random selection of each duration within each session. This included sessions using four different SDs (probe vSD#1: 1, 2, 3 and 4 s; probe vSD#2: 0.25, 0.5, 0.75 and 1 s) and sessions using three different SDs (probe vSD#3: 1, 2 and 3 s; probe vSD#4: 1, 3 and 5 s). Animals were tested for three sessions on each of the four vSD probes and presented data represent the mean of these three sessions. Animals were also assessed using probe test where the SD was fixed and changed across session (probe fixed SD: 1 and 5 s; four sessions of each probe) to assess if the observed phenotype in the vSD probes were related to the unpredictability of the SDs. In all SD probes, the LH was 0.5 s longer than the longest SD. All other task parameters remained constant and identical to the baseline rCPT procedure.

##### Manipulating target probability

In this probe, the target probability was reduced from 50% to 30% between sessions to increase the demand on behavioural inhibition and attention when the target stimulus is less frequently presented (Berwid et al., 2005; Rose et al., 2001). Animals were tested for five sessions with an SD of 2.5 s.

##### Manipulating ITI

In this probe, the ITI increased from 2 to 4 s between sessions based on the prediction that longer ITIs challenge behavioural inhibition by extending the time period during which the withholding of responding is required (Conners et al., 2003; Epstein et al., 2007; Hervey et al., 2006; Rose et al., 2001). Animals were tested for four sessions with an SD of 2.5 s.

##### Manipulating session length

In this probe, the session length was extended from 45 to 90 min. Animals were tested for four sessions with an SD of 1 s. An extended session probe was administered to assess whether ACC-lesioned and sham mice differ in their ability to maintain rCPT performance when required to engage in the task for a longer period of time (Stroh, 1971).

##### Distractors

In this probe, the central test stimulus was flanked by two identical stimuli of an either congruent (the same reward contingency as the test stimulus) or incongruent (different reward contingency as the test stimulus) nature with the rational that distractors introduce noise and impair performance (Eriksen and Eriksen, 1974; Kim et al., 2015). Responding to the distractor stimuli was without consequence. Within each session, one third of trials were presented with congruent distractors, one third of trials were presented with incongruent distractors and one third of trials were within-session non-distractor control trials. The distractor probe was administered with three different SDs varied across sessions (Distractors#1: 4 s SD, Distractors#2: 2.5 s SD, Distractors#3: 1 s SD). Each SD was presented for three sessions. All other task parameters remained constant to the baseline rCPT.

### Histology

At completion of behavioural testing, animals were terminally anaesthetised with sodium pentobarbital (Dolethal, Vetoquinol, UK) and perfused transcranially with 0.01 M PBS followed by 4% paraformaldehyde (PFA) in PBS. Brains were post-fixed in 4% PFA, immersed in 30% sucrose, and frontal cortical sections were sliced in 60 µm coronal sections. Slices were stained with Cresyl violet prior to immersion in descending concentrations of ethanol followed by xylene and mounting media. All sections were assessed and lesion extents were drawn according to a standard mouse brain atlas (Paxinos and Franklin, 2007).

### Data analysis and statistics

In the rCPT, a response to the target stimulus (S+) was scored as a *hit*, failure to respond to the target stimulus was scored as a *miss*, withholding from responding to a non-target (S–) was scored as a *correct rejection* and responding to a non-target was scored as a *false alarm*. For each animal, *hit rate* (HR) was calculated as the number of hits as the ratio of the total number of S+ presentations. *False alarm rate* (FAR) was calculated as the number of false alarms as the ratio of the total number of S– presentations. Performances were also evaluated by signal detection measures’ discriminative sensitive (d′) and response bias (c) derived from FAR and HR. The discrimination sensitivity index d′ was calculated as in Macmillan and Creelman (2004)

$$d^{\prime }=z\left(hit\;rate\right)-z\left(false\;alarm\;rate\right)$$

with higher values showing a preference for responding to the target stimulus relative to non-target stimuli. The response criterion was calculated as

c=−0.5(z(hitrate)+(z(falsealarmrate))

with larger c values indicating fewer responses to both the target and non-target stimuli. Correction trials (whereby a response to a non-target stimulus was always followed by another non-target stimulus trial) were included in all analysed data. Response latencies and reward retrieval latencies could not be analysed due to loss of data. Performances in the baseline rCPT was analysed by one-way analysis of variance (ANOVA) with lesion group as the between-subject variable. Performances in the rCPT probe tests were analysed by two-way repeated-measures ANOVAs with lesion group as the between-subject variable and probe manipulation (SD, target probability, ITI, session length or distractor condition) as the within-subject variable. The data from the probe tests of both ITI and target probability was compared to the mean performance on four baseline sessions where target probability was 50%, and the ITI was 2s. For the session length probe, the 1-s fixed SD day was used as the control condition. All analyses were done using SPSS (v22.0, IBM Corp., Armonk, NY, USA).

## Results

### Histology

**Figure 1.**
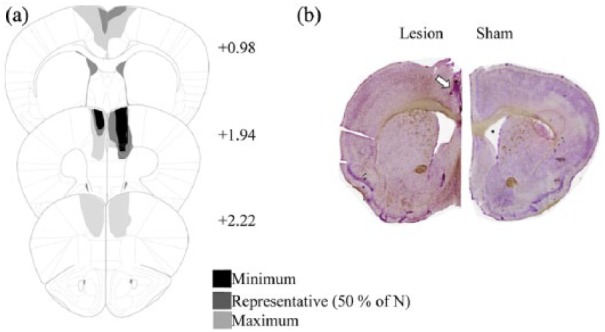
Schematic drawings (a) and representative photomicrographs (b) of the lesions and sham controls. (a) Light shading represents the largest damage observed at that coronal section (measured as distance in millimetre from bregma), black shading represents minimum damage and dark grey represents an animal with typical damage. Drawings adapted from Paxinos and Franklin (2007). (b) Photographs of coronal sections of a representative lesion (left side) and sham animal (right side). The white arrows indicate lesions.

See Figure 1(a) and (b) for representative photomicrographs and schematic drawings of the lesioned group. No sham animals showed any damage beyond expected needle tracts. Damage in the lesioned group generally did not extend beyond two sequential 60 µm thick sections (with 720 µm distance between each collected section). The extent of damage along the anterior–posterior axis was restricted to AP –2.20 and AP –0.98, and was centred on cingulate cortex area 1 (Cg1). In three animals, damage extended ventrally into the prelimbic cortex. All lesioned animals had some damage to overlaying cortex, mainly secondary motor cortex, with three lesioned mice showing limited damage to primary motor cortex.

### Post-surgery: baseline rCPT

There were no differences between groups in pre-surgery performance (data not shown). Sham and lesioned mice did not differ in sessions taken to recover to pre-surgery performance levels in the baseline rCPT (F_1,20_ = 0.764, p = 0.392; sham M:6.50, standard deviation (STDEV) = 5.53; lesion M: 8.75, STDEV = 6.30). For the last 2 days of baseline rCPT testing using a 4-s SD, performance between the lesion and sham group was equivalent for HR (F_1,20_ = 0.573, p = 0.458), d′ (F_1,20_ = 0.617, p = 0.441), c (F_1,20_ = 1.486, p = 0.237) and FAR (F_1,20_ = 1.949, p = 0.178). However, lesioned animals continued to make significantly more ITI touches (responses to the screen during the inter-trial interval) than sham controls (Table 2; F_1,20_ = 7.612, p = 0.012).

### Post-surgery probe tests

#### vSD

**Figure 2.**
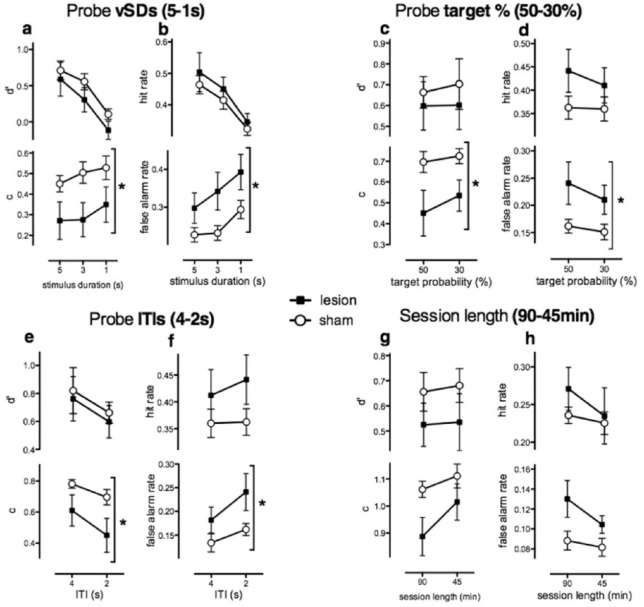
Performance of ACC-lesioned and sham controls in the rCPT when challenged with tests of variable stimulus durations (vSD; a, b), target probabilities (c, d), inter-trial intervals (e, f) and session length (g, h). Data are presented as mean ± SEM values. ACC-lesioned animals showed significantly reduced response criterion c and significantly increased false alarm rates compared to sham mice in tests of vSD, target probabilities and inter-trial intervals. ACC-lesioned animals also tended to show reduced c and increased FAR in tests of session length. Asterisks denote significant main effect of group at p < 0.05. c: response criterion; d′: discrimination sensitivity; SD: stimulus duration; vSD: variable stimulus durations; ITIs: inter-trial intervals.

Lesioned animals showed decreased values of the c parameter and increased FAR when vSD were introduced. When using vSD (1, 3 and 5 s), lesioned animals showed an SD-independent decrease in response criterion (Figure 2(a); group: F_1,18_ = 5.973, p = 0.025; group × SD: F_2,36_ = 0.204, p = 0.816) and increased FAR (Figure 2(b); group: F_1,18_ = 6.433, p = 0.021; group × SD: F_2,36_ = 0.489, p = 0.617) relative to sham controls. Lesioned animals also made more ITI touches (Table 2; F_2,36_ = 5.141, p = 0.035). The lesion group showed no changes in d′ (Figure 2(a); group: F_1,18_ = 1.222, p = 0.284; group × SD: F_2,36_ = 0.371, p = 0.392) or HR (Figure 2(b); group: F_1,18_ = 0.529, p = 0.476; group × SD: F_2,36_ = 0.074, p = 0.929).

Shorter SDs were associated with reduced d′ (F_2,36_ = 37.212, p < 0.0001), FAR (F_2,36_ = 7.871, p < 0.0001) and HR (F_2,36_ = 30.623, p < 0.0001). There was no effect of SD on c (F_2,36_ = 1.6570, p = 0.222). The lesioned group showed the same behavioural profile when using alternative ranges of vSD, and lower fixed SD (2.5 s), with the exception of short vSD (1–0.25 s) which introduced floor effects in both groups (Table 1).

#### Target probability

When reducing the target probability, lesioned animals showed a probability-independent decrease in response criterion (Figure 2(c); group: F_1,20_ = 6.501, p = 0.019; group × probability: F_1,20_ = 0.778, p = 0.388) and a probability-independent increase in FAR (Figure 2(d); group: F_1,20_ = 6.176, p = 0.022; group × probability: F_1,20_ = 0.521, p = 0.479). Lesions did not affect HR (Figure 2(d); group: F_1,20_ = 2.069, p = 0.166; group × probability: F_1,20_ = 1.549, p = 0.228) or d′ (group: F_1,20_ = 0.321, p = 0.578; group × probability: F_1,20_ = 0.076, p = 0.786). Target probability had no effect on HR, FAR, c, d′or ITI touches (Figure 2(c and d); all ps ≥ 0.084).

#### ITIs

When the event rate of the session was slowed by prolonging the ITI from 2 to 4 s, lesioned animals showed an ITI-independent decrease in response criterion (Figure 2(e); group: F_1,20_ = 5.653, p = 0.028; group × ITI: F_1,20_ = 1.016, p = 0.325) and an ITI-independent increase in FAR (Figure 2(f); group: F_1,20_ = 4.576, p = 0.045; group × ITI: F_1,20_ = 1.018, p = 0.325). There was no effect of group on HR (Figure 2(f); group: F_1,20_ = 1.973, p = 0.176; group × ITI: F_1,20_ = 0.845, p = 0.369) or d′ (Figure 2(e); group: F_1,20_ = 0.119, p = 0.734; group × ITI: F_1,20_ = 0.001, p = 0.975). The longer ITI caused a decrease in c (F_1,20_ = 10.298, p = 0.004) and an increase in FAR (F_1,20_ = 6.836, p = 0.017) without affecting d′ (F_1,20_ = 2.945, p = 0.102), HR (F_1,20_ = 1.139, p = 0.299) or ITI touches (F_1,20_ = 0.387, p = 0.541) (Figure 2 (e and f)).

#### Session length

When comparing the 90-min session to the baseline 45-min session, there were near-significant main effects of lesion on c (Figure 2(g); group: F_1,18_ = 3.889, p = 0.064; group × session length: F_1,18_ = 1.765, p = 0.201) and FAR (Figure 2(h); group: F_1,18_ = 4.119, p = 0.057; group × session length: F_1,18_ = 1.612, p = 0.220). In the 90-min session, lesioned mice made more ITI touches than sham mice (Table 2; F_1,18_ = 8.815, p = 0.008). There was no effect on HR (Figure 2(h); group: F_1,18_ = 0.702, p = 0.413; group × session length: F_1,18_ = 1.585, p = 0.224) or d′ (Figure 2(g); group: F_1,18_ = 1.162, p = 0.295; group × session length: F_1,18_ = 0.060, p = 0.810).

#### Flanking distractors

**Figure 3.**
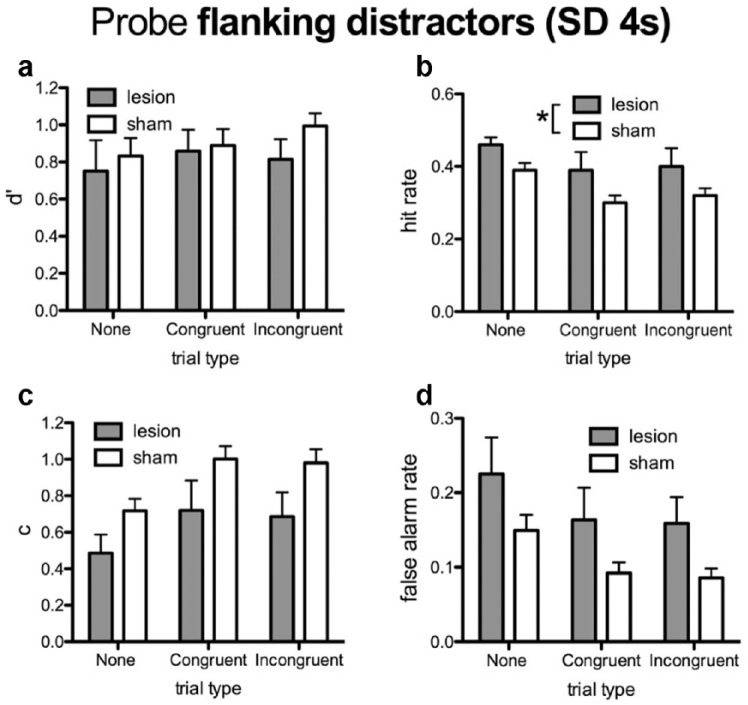
Performance of ACC-lesioned and sham controls in the rCPT when challenged with flanking congruent or incongruent distractors. Data are presented as mean ± SEM values. ACC-lesioned animals showed significantly higher hit rate and a general tendency for increased false alarm rate and lower response criterion compared to sham mice. The presence of distractors significantly reduced the hit rate and false alarm rate in both groups. Asterisks denote significant main effect of group at p < 0.05. c: response criterion; d′: discrimination sensitivity.

When introducing distractors (using a 4-s SD), there were trends for a distractor-independent decrease in c (Figure 3(a); group: F_1,20_ = 4.288, p = 0.052; group × trial type: F_1,20_ = 0.377, p = 0.688) and distractor-independent increase in FAR (Figure 3(b); group: F_1,20_ = 4.019, p = 0.059; group × trial type: F_1,20_ = 0.018, p = 0.982) in lesioned animals. Lesioned animals had significantly higher HRs than sham controls (Figure 3(d); group: F_1,20_ = 4.859, p = 0.039; group × trial type: F_1,20_ = 0.327, p = 0.723), but no effect on d′ (group: F_1,20_ = 0.605, p = 0.446; group × trial type: F_1,20_ = 0.564, p = 0.584). On trials that included distractors, animals showed decreased FAR (F_2,40_ = 21.241, p < 0.0001) and decreased HR (F_2,40_ = 10.372, p < 0.0001). Distractors did not affect d′ (F_2,40_ = 1.282, p = 0.289). There were no significant differences in performance on congruent versus incongruent distractor trials. The data from distractor trials with 2.5 or 1 s SD are summarised in Table 1.

## Discussion

We have assessed whether lesion disruption of the ACC impacts performance in a recently developed touchscreen rodent task that closely mimics widely used human CPT procedures We validate the task for cross-species translational studies by showing that damage to the ACC of the mouse prefrontal cortex produces a more liberal response criterion resulting from increased FARs together with modest increases in responding to target stimuli, as well as increased ITI responses. Lesions were without effect on attentional function as measured by discriminative sensitivity d′. This behavioural phenotype was consistent throughout rCPT testing and was observed most robustly when task parameters were set to increase task difficulty. The data are in general agreement with studies implicating the anterior cingulate in error detection and suppression of inappropriate responses and indicate that the rCPT may be useful as a translational measure of fronto-executive function.

The anterior cingulate has been implicated in various supporting functions in executive control (Corbetta and Shulman, 2002; Posner and Petersen, 1990). In human experimental studies, such functions consistently consist of processing of error signals and response inhibition. In CPTs and Go/No-Go tasks, lesions encompassing anterior frontal regions are associated with a more liberal response criterion and increased FARs (Glosser and Goodglass, 1990; Salmaso and Denes, 1982). Neuroimaging and electrophysiological studies show that false alarm errors consistently activate the anterior cingulate (Carter et al., 1998; Rubia et al., 2001). The false alarm-related ACC activity is stronger than the activity following correct responses or following correct inhibitions (Braver et al., 2001; Hester, 2004), which may support adjustments such as speed/accuracy trade-off and behavioural remedial actions following inappropriate responses (Gehring and Knight, 2000; Pailing et al., 2002; Scheffers et al., 1996). Furthermore, measures of event-related potentials using cued CPTs show increased ACC activity prior to non-target trials relative to target trials (Fallgatter et al., 2002) implicating the region in response inhibition and the mediation of an internal representation of ‘don’t respond’ (Braver et al., 2001). Aberrant structural and error-related anterior cingulate activity may also contribute to impairments in response inhibition tasks in mental health disorders such as attention deficit hyperactivity disorder (ADHD; Rubia et al., 2001), obsessive-compulsive disorder (OCD; Fitzgerald et al., 2005; Gehring and Knight, 2000), schizophrenia (Fallgatter et al., 2003; Salgado-Pineda et al., 2004), dementia (Sanchez-Castaneda, 2009) and drug abuse (Forman et al., 2004; Hester and Garavan, 2004; Leland et al., 2008). The observation of lower response criterion and increased FARs in ACC-lesioned animals is in broad agreement with such human studies and suggests some cross-species functional homology in the mouse.

The response profile of ACC-lesioned animals is also in general agreement with data from 5- and 3-serial reaction time tasks (CSRTT) that demonstrate the importance of the integrity of, and balanced transmission in, the ACC for inhibitory response control and the processing of incorrect responses. In the 5-CSRTT, consistent with the current data, anterior cingulate lesions in the rat can cause selective impulsive-like increases in premature responding without affecting discriminative sensitivity in the 5-CSRTT (Muir et al., 1996), although a chemogenetic silencing of the dorsal ACC in mice did not alter response control in the same task (Koike et al., 2016). High-impulsive rats also show increased dopamine turnover (Dalley et al., 2002), decreased γ-Aminobutyric acid (GABA) binding (Jupp et al., 2013) and decreased metabolic activity in the anterior cingulate regions as measured by [^14^C]deoxyglucose (DG) uptake (Barbelivien et al., 2001), and intra-ACC glutamic acid decarboxylase (GAD) inhibition selectively increases premature responses in the three-choice serial reaction time task (Pehrson et al., 2013). Electrophysiological recordings in the rat show, like humans, increased ACC activity prior to stimulus onset and following incorrect responses (Totah et al., 2009) as well as altered ACC-prelimbic synchrony prior to stimulus onset (Totah et al., 2013). Notably, the behavioural profile of pharmacological animal models of psychiatric disorders includes comparable deficits in inhibitory response control. This includes rats subchronically treated with phencyclidine (PCP) in the 5C-CPT procedure (Barnes et al., 2012), repeated amphetamine administration in the sustained attention task (SAT) (Deller and Sarter, 1998), systemic N-methyl-D-aspartate (NMDA) antagonist treatment in the 5-CSRTT (Amitai et al., 2007; Paine and Carlezon, 2009) and the MAM-E17 model in the rCPT (Mar et al., 2017), which are all associated with increased false alarm errors. Here, we demonstrate that disinhibitory behavioural effects of ACC lesioning are also detected in the rCPT, indicating that the task is a valid approach for studying prefrontal function in the mouse that is of psychiatric relevance.

Yet ACC lesioning did not cause apparent effects on attention as defined as changed in discrimination sensitivity. The phenotype was characterised by a decrease in response criterion driven primarily by a consistent, significant increase in the FAR, with smaller increases in HR that were significant only on select probes (decreasing SDs or with flanking distractors). The increase in ITI responses also points to a general disinhibitory effect of ACC dysfunction on the rCPT. The lack of interactions between lesions and attentional difficulty of the probe tests also suggests that the phenotype is unrelated to attention. In a parallel effort to examine the functional heterogeneity of the rat medial prefrontal cortex (mPFC) on rCPT (Fisher et al., unpublished), ACC-lesioned rats showed only a transient decrease in discrimination sensitivity, with no indication of impaired inhibitory control. Both rat and mouse ACC lesions leaving discrimination sensitivity largely unchanged suggest that the ACC is not critical for attentional functioning as measured by rCPT. In the 5-CSRTT, a test of visuospatial stimulus detection and response inhibition, some rat studies have observed impairments in discriminatory sensitivity following ACC lesions (Chudasama et al., 2003; Passetti et al., 2002), but these lesions included dorsal prelimbic cortex, and an ACC-restricted lesion failed to impair accuracy (Muir et al., 1996). Pharmacological, optogenetic and chemogenetic manipulations of the ACC have observed attentional disruptions on the 3- or 5-CSRTT, however, in mice and rats (Kim et al., 2015; Koike et al., 2016; Pehrson et al., 2013), suggesting that the 5-CSRTT and the rCPT are sensitive to different deficits in performance following ACC damage and may offer a complimentary function when assessing attentional and response control. This, in combination with the consistent way in which the current data support the human literature on ACC and response control, highlights the importance of behavioural tasks with high cross-species translational value.

In the rCPT, lesions of the rat medial prefrontal cortex, including prelimbic and infralimbic subregions, impaired discrimination sensitivity (d′) on baseline rCPT (Mar et al., unpublished). More specific prelimbic cortex lesions produced d′reductions in probes where SD was reduced or the event rate was high (Fisher et al., unpublished). Together, these results suggest that this area is more critical for attentional processing in this task than the ACC. In support of this, Granon (1998) found prelimbic cortex (PL) lesions in the rat to disrupt a brightness-discrimination-based CPT in rats but not impair two-choice serial reaction time task performance, pointing to a distinct role for PL function in sustained attention. Passetti et al. (2002) found that, by manipulating ITIs, PL-ACC lesions disrupt the temporal sequencing of visuospatial responding and that this may also cause accuracy impairments in the 5-CSRTT. A further possibility is that ACC dysfunction can impair divided detection, which possibly could serve to leave focused attention intact (Lashley, 1931; Petruno et al., 2013; Pöppel et al., 1973; Stoerig et al., 1985). The anterior cingulate exhibits heterogeneity in its regional organisation, and hence possibly its functioning, in both humans (Braver et al., 2001; Kiehl et al., 2000; Menon et al., 2001) and rodents (Delatour and Gisquet-Verrier, 2001; Heidbreder and Groenewegen, 2003), which may account for some of the inconsistent effects of ACC dysfunction on discriminative sensitivity.

As well as response impulsivity, the functional heterogeneity of the ACC could support associative learning and coding unsigned prediction errors (Bussey et al., 1997; Cardinal et al., 2003; Hayden et al., 2011), memory (Cabeza et al., 1997; Frankland et al., 2004; Petit et al., 1998; Tang et al., 2005), motor coordination (Paus et al., 1993; Procyk et al., 2000) and novelty detection (Clark et al., 2000). However, there is little to suggest that the response disinhibitory effects of the ACC lesion derive from impairments in domains such as motoric function, learning and memory or novelty processing per se as (a) the lesion did not affect discrimination sensitivity – hence memory as well as attention is unaffected; (b) the deficits were not present on baseline, fixed SD trials, indicating that motoric functions and alertness were not directly affected; and (c) animals were well trained on the task and pre-exposed to the probe tests before lesioning, which minimised any learning and novelty effects on performance. Lesions also did not affect re-learning of the task post-surgery.

In addition to a role of the ACC in response impulsivity (inability to withhold a response), the area has been implicated in choice impulsivity (impulsive decision making; Winstanley et al., 2006). The ACC regulates the amount of effort rats are willing to invest in order to obtain a reward (Rudebeck et al., 2006), with dorsal ACC-lesioned rats preferring low-cost, low-reward options over the high-cost, high-reward alternative selected by shams (Walton et al., 2003). Although the rCPT is not specifically designed to assess choice impulsivity (e.g. there is no more physical effort associated with responding to a target than a non-target), it seems unlikely that an impairment in choice impulsivity would result in the pattern of performance impairment observed in the current study, most consistently being an increase in the FAR, a response profile that is more in keeping with impulsive response than impulsive choice. The lowest cost option, no response, is not chosen more often by lesioned mice than shams.

The secondary motor cortex (M2) has been shown to support performances in a temporal discounting procedure, with localised GABA agonists introducing cross-trial variability in the capacity to wait for large, delayed rewards (Murakami et al., 2017). The ACC-lesioned mice in the current study all showed some damage to M2 (roughly a sixth of the total M2 volume on average), raising the possibility that the behavioural effects are produced by damage to ACC and/or M2. However, in the study by Murakami et al. (2017), M2 inactivation was found to introduce both increased and decreased waiting times in rats, which is different from the consistently disinhibited profile observed in the current study. Moreover, M2 was shown to support delay discounting (Murakami et al., 2017), and the rCPT has no obvious discounting component; non-target trials in the rCPT represent no reward and responses to non-target results in further delay in the opportunity to obtain any reward. Under these current conditions, the ACC has repeatedly been found to be critical (e.g. Barbeliven et al., 2001; Dalley et al., 2002; Jupp et al., 2013; Muir et al., 1996; Pehrson et al., 2013).

The introduction of flanking distractors disrupted the performance of both groups through general reductions in responding; distractors increased the response criterion c through decreasing hit and FARs. The higher HR of lesioned mice compared to sham mice in one distractor probe could, in the absence of other significant differences, be interpreted as an improvement in attention. When seen in light of the pattern of results across the study, as well as the numerically higher values of responding in lesioned mice in general, the increase in HR seems more in line with a general disinhibited response profile. In agreement with a previous rCPT study with mice (Kim et al., 2015), there were no congruency effects, and the inclusion of distractors did not affect d′. This is in contrast to the pattern of responding of rats with mPFC lesions on rCPT, as well as several different pharmacological rat models, where congruent and incongruent distractors numerically improved and impaired performance, respectively, in comparison to non-distractor trials, with no change in the overall level of responding (Mar et al., unpublished; Fisher et al., unpublished; Mar et al., 2017). The rat data are in line with human studies of sustained attention using flanker tasks (Eriksen, 1995). The reductions in responding in mice may be due to animals interacting with the distractors themselves, rather than the responsive stimuli at the centre of the screen (Kim et al., 2015). In this view, the distractors work excessively well in mice in that animals are distracted from responding to the central stimulus altogether. Ongoing work is addressing this possibility with the aim of developing distractors that can disrupt attention and inhibitory control in mice.

## Conclusion

Human performance on CPTs is reliant on activity in the ACC for the detection of false alarm errors and response inhibition on non-target trials. In broad agreement with such studies, lesions centred on the anterior cingulate in the mouse produced impairments in inhibitory response control as assessed by the touchscreen rCPT. This suggests that the rCPT has validity for assessing prefrontal cortical–dependent functions in the mouse and may have the capability of providing meaningful translationally relevant links between animal and human cognition.
